# Dynamics of faecal egg count in natural infection of *Haemonchus spp*. in Indian goats

**DOI:** 10.14202/vetworld.2015.38-41

**Published:** 2015-01-09

**Authors:** Nimisha Agrawal, Dinesh Kumar Sharma, Ajoy Mandal, Pramod Kumar Rout, Yogendra Kumar Kushwah

**Affiliations:** 1 Division of Goat Health and Goat Genetics and Breeding, Central Institute for Research on Goats, Mathura, Uttar Pradesh, India; 2 Animal Breeding Section, Eastern Regional Station, National Dairy Research Institute, Nadia, West Bengal, India

**Keywords:** goats, *Haemonchus*, faecal egg count

## Abstract

**Aim:**

Dynamics of faecal egg count (FEC) in *Haemonchus* spp. infected goats of two Indian goat breeds, Jamunapari and Sirohi, in natural conditions was studied and effects of genetic and non-genetic factors were determined.

**Materials and Methods:**

A total of 1399 faecal samples of goats of Jamunapari and Sirohi breeds, maintained at CIRG, Makhdoom, Mathura, India and naturally infected with *Haemonchus* spp., were processed and FEC was performed. Raw data generated on FEC were transformed by log_e_ (FEC+100) and transformed data (least squares mean of FEC [LFEC]) were analyzed using a mixed model least squares analysis for fitting constant. Fixed effects such as breed, physiological status, season and year of sampling and breed × physiological states interaction were used.

**Result:**

The incidence of *Haemomchus* spp. infection in Jamunapari and Sirohi does was 63.01 and 47.06%, respectively. The mean LFEC of both Jamunapari and Sirohi (does) at different physiological stages, namely dry, early pregnant, late pregnant early lactating and late lactating stages were compared. Breed, season and year of sampling had a significant effect on FEC in *Haemomchus* spp. infection. Effect of breed × physiological interaction was also significant. The late pregnant does of both breeds had higher FEC when compared to does in other stages.

**Conclusion:**

Breed difference in FEC was more pronounced at the time of post kidding (early lactation) when sharp change in FEC was observed.

## Introduction

Faecal egg count (FEC) in gastrointestinal nematode infection represents the severity of disease as well as the immune status of the animal. However, dynamics of FEC varies in different seasons, physiological states, breeds, age, and managements and also in different geographic area [1]. Relaxation of immunity in reproducing females also affects FEC that results in peri-parturient period. The peri-parturient rise (PPR) in FEC is important because it increases pasture contamination at the time of parturition that in turn greatly increases the risk of infection in the very susceptible young ones. The marked increase in susceptibility of lactating animals to gastrointestinal (GI) nematode infection had been extensively documented in sheep; however, it lack evidences in goats. PPR in FEC has been reported in lactating Galla does and Small East African goats breed in Kenya [2], dairy goats in The Netherland [3], indigenous goats in Brazil [4]. The stress of high level of milk production was associated with increased susceptibility in alpine dairy goats to GI nematode in France [5]. Some resistant sheep breeds like St. Croix and Florida Native certainly had a lower PPR than temperate breeds like Rambouillet or dorset × Rambouillet [6].

Reports on dynamics of FEC, especially during peri-parturient period and other physiological states in Indian sheep and goat breeds are meager [7-10]. Therefore, the objective of the present study was the dynamics of FEC in two goat breeds in natural infection of *Haemomchus* spp. in different physiological states under semi-intensive management system.

## Material and Methods

### Ethical approval

All essential procedures of sample collections were performed strictly as specified by Institutional Ethics Committee with minimal stress to animals.

### Location of study

The study was conducted at the Central Institute for Research on Goats (CIRG), Makhdoom, Farah, Mathura, Uttar Pradesh, India, located at 27°29’ North latitude and 77°40’ East latitude (msl-174 m). The climate of the study area is classified as tropical (semi-arid zone). Temperature varies from 21°C to 42°C in summer and 6°C to 30°C in winter (both average). The annual rainfall is about 750 mm and scattered mainly during June-September. Winter is generally dry and cold. The soil is sandy, and vegetation is composed of natural pasture and bushes.

### Flock description and management

Jamunapari and Sirohi does in different physiological states *viz*. pregnant, lactating and dry maintained at Central Institute for Research on Goats, Makhdoom, Mathura (India) were used for the study. In brief, Jamunapari is a large sized goat known for its milk production [11]. It is native of Chakarnagar area of Etawah district of Uttar Pradesh, India. The average daily milk production varies from 1 to 2.5 L. Average lactation length was of 151.42±10.92 days. The Sirohi, one of the important defined breeds of India, is native of Rajasthan. The lactation length averaged as 167.5 days under semi-intensive system [12]. Being breeding flocks, animals of both flocks were bred as per breeding schedule. Two breeding seasons were planned every year resulting two pregnancy periods of 5 months each i.e. May-October and October-March. As per farm schedule, the animals of all categories were maintained separately in semi-intensive system of management. They were provided concentrate ration with mineral mixture i.e. 400 g/animal/day for pregnant and lactating animals and 150 g/animal/day for dry animal. All three categories were allowed 6 h grazing daily. De-worming was carried out twice annually i.e. in the pre-monsoon (June-July) and the post-monsoon (September-October) seasons. Faecal samples for parasitological examination were collected either before the anthelmintic treatment or after an interval of 4-weeks post-treatment.

### Parasitological procedure

The faecal samples were collected from the rectum of animals of both breeds, and FEC was conducted using a modified McMaster Technique [13] with each egg counted representing 200 eggs/g of faeces. Pooled faecal samples randomly collected from 5% animals (3 times) in rainy seasons were cultured for larvae to define the species composition of the nematode infecting the flocks.

### Data and experimental design

The study was conducted from August 2006 to June 2008. For study, a total of 1399 samples, 838 from Jamunapari and 561 from Sirohi, were collected and examined. The collected samples were grouped on the basis of physiological status of donor *viz*. dry (non-pregnant, non-lactating), pregnant and lactating. Samples from pregnant does were subdivided into early (in 4^th^ month of gestation) and late (15-30 days before kidding) pregnant stages. Similarly, faecal samples from lactating does were also divided into early (1^st^ month) and late (3^rd^ month) lactation to study the variation in FEC more discreetly. Thus, FEC of adult females was monitored at five different stages such as dry, early pregnant, late pregnant, early lactating and late lactating periods. Faecal samples collection was, however, either before the anthelmintic treatment or 4-weeks post-treatment.

Similarly, observations of FEC were also analyzed on seasons (summer, rainy, winter) basis.

### Statistical analysis

Data generated on FEC was Log-transformed (log_e_ [FEC+100]) to normalize. Normalized data were analyzed statistically through least squares ­analysis [14]. The statistical models applied included only fixed effects, and effects fitted varied with sampling periods. The fixed effects were Breed (2 classes), physiological states of goats (5 classes: dry, early pregnant, late pregnant, early lactating and late lactating), seasons (3 classes), year (3 classes). The dependent variables analyzed were FEC.

To determine the effects of various factors on FEC in different physiological stages following model was used:


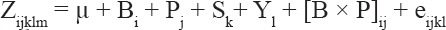


Where,

Z_ijlm_ = is the m^th^ observation of individuals, µ = Overall mean, B_i_ = Fixed effect of i^th^ breed [i = 1, 2], P_j_ = Fixed effect of j^th^ physiological conditions [j= 1, 2,….5], S_k_ = Fixed effect of k^th^ season of sampling [k = 1, 2, 3], Y_l_ = Fixed effect of l^th^ year of sampling [l = 1, 2, 3], [B × P]_ij_ = Interaction effect between i^th^ breed and j^th^ physiological condition, e_ij_ = Random error associated with observation with mean 0 [zero] and variance δ^2^ e.

The mean values resulted from the analysis were reverse transformed and presented in modified mean as geometric means of FEC (GFEC). The comparison of different sub-groups means was made by Duncan’s multiple range test as described by Kramer [15].

## Results and Discussion

In this climatic zone, the major source of parasitic infection in animals was from pasture. The morphological differentiation of larvae, emerged out from bulk cultures for each sampling from both the breeds, showed that the predominant larvae were of *Haemonchus contortus*. Other GI nematodes like *Oesophagostomum* spp. (1.66%), *Strongyloides* spp. (2.00%) and *Trichuris* spp. (1.33%) were also recorded, however, they were sporadically occurring. Previous reports from this area also describe *Haemonchus* spp. as the predominant strongyle infection in semi-arid zone of India [9,16,17]. The overall prevalence rate of *Haemonchus* spp. infection in the flocks under the study was 63.01% (i.e. 528/838) for Jamunapari and 47.06% (i.e. 264/561) for Sirohi does.

The moderate incidence of parasitism observed in the present study was similar as has been reported from semi-arid region of India [18] and some other part of the world [19].

**Figure-1:**
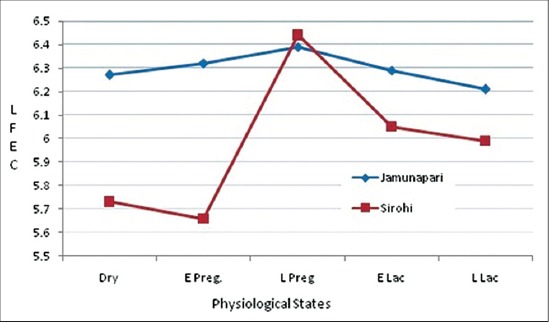
Peri-parturient rise (faecal egg count) in Jamunapari and Sirohi Does.

Least squares mean of FEC (LFEC) and back transformed values of GFEC in different physiological stages of both breeds are shown in Table-1. In the present study, breeds had significant (p<0.01) effect on FEC and Sirohi does have significantly lower FEC values than Jamunapari does. The effect of physiological state of animal on FEC was also statistically significant. Though, FEC in dry does and early pregnant could not show significant difference, the mean LFEC of late pregnant does was significantly higher than the corresponding values in dry does (p<0.05) (Table-1). Subsequently LFEC started receding till late lactation stage and further to maintain its dry period state in both breeds. The breed × physiological status interaction was significant (p<0.05) for FEC. There was a different pattern of the peri-parturient change in two breeds. In Jamunapari goats, there was no significant change observed in FEC among pregnant, dry, and lactating goats. Our findings corroborated with the findings of Chauhan *et al*. [8] who reported no significant difference between dry, pregnant, and lactating does of the same breed. In Sirohi, up to early pregnant stage change in mean LFEC was not significant. However, mean LFEC in late pregnant and early lactating Sirohi was significantly higher (p<0.05) than dry and early pregnant animals [19]. No significant change in FEC between late pregnant and lactating Sirohi does was same as in Jakhrana, a native goat breed of semi-arid Rajasthan [9]. In Sirohi, changes in FEC were more apparent than in Jamunapari. Both breeds showed highest LFEC in late pregnancy similar to Gibbs and Barger [20] who found peak egg counts just before lambing. However, after kidding there was a decline in FEC (Figure-1). Reduction in FEC from late pregnant to early lactation stage might be due to farm management practices, where the pre-kidding de-worming was necessary. However, this reduction was less apparent in Jamunapari goats may be attributed to their higher milk production as compared to Sirohi does (Figure-1). Hoste and Chartier [5] stated that the stress of high level of milk production in Alpine dairy goats (France) was associated with increased susceptibility of GI nematodes. Similarly, Baker *et al*. [2] reported more marked and persistent PPR in FEC in Galla (high milk producing breed) than SEA goats. Also, there is report that systemic immunity began to wane during late pregnancy [21]. The difference in mean LFEC can be attributed to the difference in grazing habit or breed difference.

**Table-1:** Overall LSM of faecal egg counts in Jamunapari and Sirohi goats in different physiological stages*.

Source of variation	No.	LFEC (GFEC/g of faeces)
Overall mean of animals	1399	6.14±0.05 (364)
Breed		
Jamunapari	838	6.30±0.05^a^ (444)
Sirohi	561	5.97±0.07^b^ (291)
Physiological stages		
Dry	731	6.00±0.05^a^ (303)
Early pregnant	122	5.99±0.13^a^ (299)
Late pregnant	194	6.41±0.10^b^ (507)
Early lactation	170	6.17±0.12^ab^ (378)
Late lactation	182	6.10±0.10^a^ (345)
Season of collection		
Summer	452	6.16±0.08^a^ (373)
Rainy	413	6.70±0.08^b^ (712)
Winter	534	5.54±0.07^c^ (154)
Year of collection		
2006	432	5.71±0.07^a^ (201)
2007	657	6.08±0.06^b^ (337)
2008	310	6.62±0.09^c^ (649)
Breed×physiological states		
Jamunapari×dry	401	6.17±0.06^a^ (378)
Jamunapari×Early pregnant	78	6.32±0.15^a^ (455)
Jamunapari×late pregnant	122	6.44±0.12^a^ (526)
Jamunapari×early lactation	133	6.34±0.13^a^[466]
Jamunapari×late lactation	104	6.21±0.11^a^ (397)
Sirohi×dry	330	5.83±0.07^a^ (240)
Sirohi×early pregnant	44	5.66±0.20^a^ (187)
Sirohi×late pregnant	72	6.39±0.16^b^ (495)
Sirohi×early lactation	37	6.00±0.22^ab^ (303)
Sirohi×late location	78	5.99±0.16^ab^ (299)

*Means with different superscripts in column under a source of variation differ significantly from each other. LFEC=Least squares mean of faecal egg count, GFEC=Geometric means faecal egg count, LSM=Least mean square

Effect of season of collection on FEC was significant (p<0.01). The highest mean LFEC was found in the rainy season, followed by summer and winter season, for both Jamunapari and Sirohi goats. These findings were in accordance with previous reports [9,16,18,19] which described maximum EPG in monsoon. However, the results were contrary to Fakae [22] who recorded incidence as high 77.8-100% with no definite seasonal distinction. The year of sampling had significant (p<0.05) effect on LFEC. This may be attributed to change in management, selection of sires and environmental conditions such as ambient temperature, humidity, rainfall, etc. Furthermore, there were reports that described the gradual increase in FECs over the gestation period, which peaked during lactation during winter and spring lambing season [23].

## Conclusion

The study revealed breed had a significant effect on FEC and so also the physiological status. PPR was sharper in Sirohi goats. In the present study, prevalence and intensity of infection was significantly higher in Jamunapari goats as compared to Sirohi.

## Author’s Contributions

NA, DKS designed the work plan, collected and processed the faecal samples for FECs. YKK and AM compiled, tabulated, transformed and analyzed the data. AM and PKR interpreted the result. DKS, AM and PKR prepared the manuscript. All authors read and approved the final manuscript.
